# Long-term follow up after denosumab treatment for osteoporosis – rebound associated with hypercalcemia, parathyroid hyperplasia, severe bone mineral density loss, and multiple fractures: a case report

**DOI:** 10.1186/s13256-020-02401-0

**Published:** 2020-08-11

**Authors:** Yves Maugars, Pascale Guillot, Joëlle Glémarec, Jean-Marie Berthelot, Benoit Le Goff, Christelle Darrieutort-Laffite

**Affiliations:** Rheumatology Department, Nantes University Hospital, 1 place Alexis Ricordeau, 44093 Nantes, Cedex France

**Keywords:** Osteoporosis, Denosumab rebound, Fracture, Hypercalcemia, Hyperparathyroidism

## Abstract

**Background:**

The rebound effect after stopping treatment with denosumab may be associated with rapid loss of the gains in bone mineral density achieved with treatment, high levels of bone remodeling markers, the occurrence of vertebral fractures, and even hypercalcemia.

**Case presentation:**

A 64-year-old osteoporotic Caucasian woman suffered from a fracture of her second lumbar vertebra in 2004. From January 2005, she was treated with denosumab for 9 years, with good densitometry results for her hip and lumbar areas, and no fractures over the last 6 years of treatment. Ten months after the treatment with denosumab was stopped, a cascade of vertebral fractures, including some in unusual locations (third thoracic vertebra), and multiple rib fractures in a context of hypercalcemia, suggested possible malignancy. A complete evaluation, including systemic, biological, and biopsy analyses, ruled out this hypothesis. The hypercalcemia was associated with normal plasma phosphate and vitamin D concentrations, and a high parathyroid hormone level, with an abnormal fixation of the lower lobe of the thyroid on sesta-methoxy-isobutyl-isonitrile scintigraphy. Histological analysis of the excised parathyroid tissue revealed hyperplasia. The associated thyroidectomy (goiter) led to the discovery of a thyroid papillary microcarcinoma.

**Conclusions:**

We consider the consequences of this rebound effect, not only in terms of the major loss of bone density (return to basal values within 3 years) and the multiple disabling fracture episodes, but also in terms of the hypercalcemia observed in association with apparently autonomous tertiary hyperparathyroidism. Several cases of spontaneous reversion have been reported in children, but the intervention in our patient precluded any assessment of the possible natural course. The discovery of an associated thyroid neoplasm appears to be fortuitous. Better understanding of the various presentations of the rebound effect after stopping treatment with denosumab would improve diagnostic management of misleading forms, as in this case. Bisphosphonates could partially prevent this rebound effect.

## Introduction

Some warning signs need to be explored and are well known after treatment with denosumab. A major rebound effect after stopping denosumab can be responsible for rapid bone loss with vertebral crushes [1–6]. Some other manifestations have been described, such as hypercalcemia in both children [7–13] and adults [14], hyperparathyroidism [15], and vertebral osteonecrosis [16]. A suspected increase in the number of cases of primary neoplasia has been reported in a recent meta-analysis [17]. We discuss a new observation with all these manifestations together, which poses diagnosis problems, and several explorations to eliminate a neoplasia. This could have been avoided with better knowledge of these rebound manifestations.

## Case presentation

**Fig. 1:**
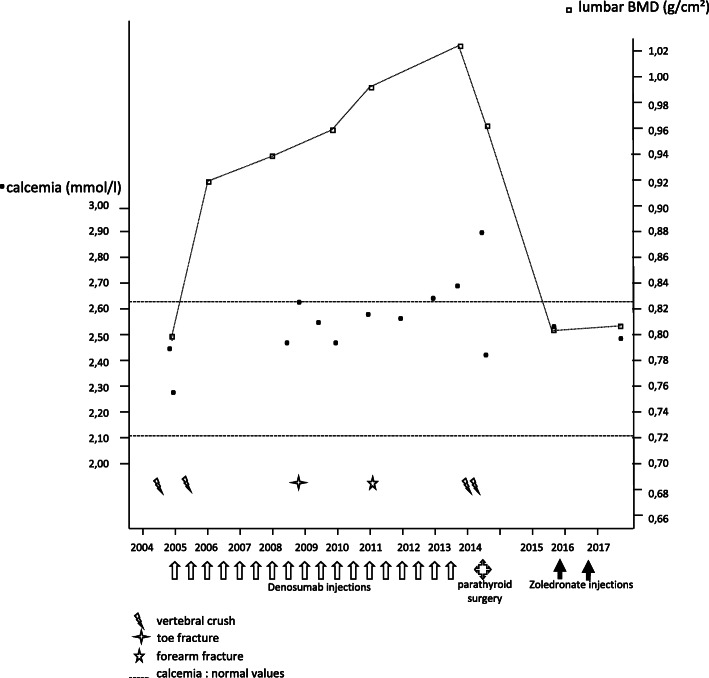
Calcemia, bone mineral density, and fracture events for a patient treated with denosumab for 9 years who experienced a rebound effect when treatment was stopped, with a combination of hypercalcemia related to hyperparathyroidism, multiple fractures, and rapid bone loss

A 64-year-old Caucasian woman suffered from a first vertebral fracture in the second lumbar vertebra (L2) in 2004 following a fall from her bicycle. She did not obtain any treatment. Dual X-ray absorptiometry (DXA) revealed osteoporosis: lumbar T-score of − 3.2 standard deviation (SD). Our patient’s characteristics during follow up are summarized in Table 1. Her phosphorus and calcium levels were normal (plasma calcium concentration = 2.50 mmol/l; normal range 2.13 to 2.65 mmol/l), parathyroid hormone (PTH) concentration was normal (48 ng/l; normal range 15 to 65 ng/l), and vitamin D level was low (13.6 ng/ml; normal range 30 to 60 ng/l). She was included in the FREEDOM protocol, comparing denosumab (60 mg, subcutaneously, every 6 months, plus 1000 mg of calcium and 800 IU of vitamin D daily) with placebo for the treatment of postmenopausal osteoporosis in January 2005. The unblinding of the trial 3 years later showed that she had been randomized to the denosumab group. Several vertebral fractures occurred during this 3-year period: fifth thoracic vertebra (T5), eighth thoracic vertebra (T8), and an aggravation of the L2 fracture. She continued to participate in the extension protocol in open mode for 6 years, and then withdrew of her own volition, with a final injection of denosumab in July 2013; there were no new vertebral fractures during this entire period. DXA in September 2013 demonstrated increased bone mineral density (BMD) of 22.3% in her lumbar region (T-score, − 1.6 SD) and of 17.0% in her total left hip (T-score, − 1.1 SD). She was a former tobacco smoker and her medical history included osteoarthritis of the knee, a hiatus hernia, hypertension, amlodipine allergy, and colonic polyps. Calcaemia monitoring revealed a return to normal values until January 2012 (2.58 nmol/l; normal range 2.13 to 2.65 mmol/l). Check-ups while our patient was still on denosumab yielded values of 2.68 nmol/l in January 2013 and 2.73 in September 2013 (Fig. 1).

**Table 1:** Patient’s characteristics during follow up

	Clinical presentation	Fractures	T-score lumbar/total hip BMD (SD)	Calcaemia (mmol/l)	Treatments
Follow up of the patient (January 2005)	Bicycle fall	L2	−3.2/−2.1	2.50	Initiation: denosumab (60 mg semi-annually), calcium (1 g daily), vitamin D (800 IU daily)
FREEDOM 3-year evaluation (January 2008)	No pain	T5, T8, and aggravation of L2	−2.2/−1.7	2.52	Pursuit: denosumab (60 mg semi-annually), calcium (1 g daily), vitamin D (800 IU daily)
FREEDOM extension (September 2013)	No pain	No new fracture	−1.6/−1.1	2.73	All treatments stopped
Hospitalization (June 2014)	Acute intense dorsal pain	T4, T9, T10, T11, L1, L3	−2.0/−1.3	2.83	None
New evaluation (September 2016)	Chronic dorsal and lumbar pain	No new fracture	−2.9/−2.3	2.47	Zoledronate (two infusions of 5 mg annually), vitamin D (100,000 IU quarterly)
New evaluation (August 2018)	Chronic lumbar pain	No new fracture	−2.9/−2.3	2.60	Treatments stopped

*BMD* bone mineral density, *L1* first lumbar vertebra, *L2* second lumbar vertebra, *L3* third lumbar vertebra, *SD* standard deviation, *T4* fourth thoracic vertebra, *T5* fifth thoracic vertebra, *T8* eighth thoracic vertebra, *T9* ninth thoracic vertebra, *T10* tenth thoracic vertebra, *T11* 11th thoracic vertebra

**Fig. 2:**
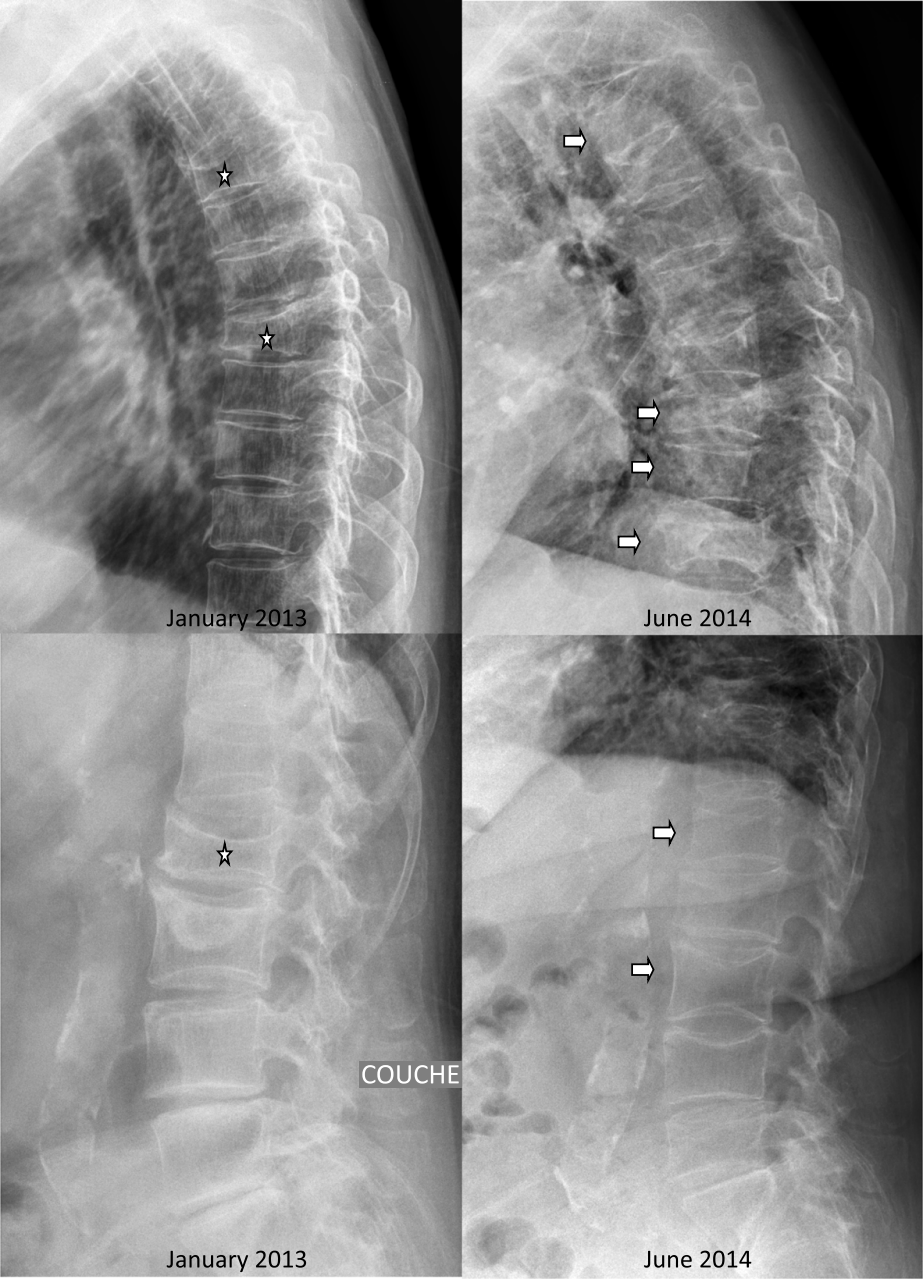
Lumbar and thoracic X-ray in January 2013, 3 months before denosumab was stopped, and in June 2014, 14 months after denosumab was stopped: three old vertebral fractures (L2 in 2004 and T5 T8 L2 (aggravation) during the period 2005-2008)(stars) and 6 new fractures (T4 T9 T10 T11 L1 L3)(arrows) with denosumab rebound. Stars are the old fractures and arrows the new one

**Fig. 3:**
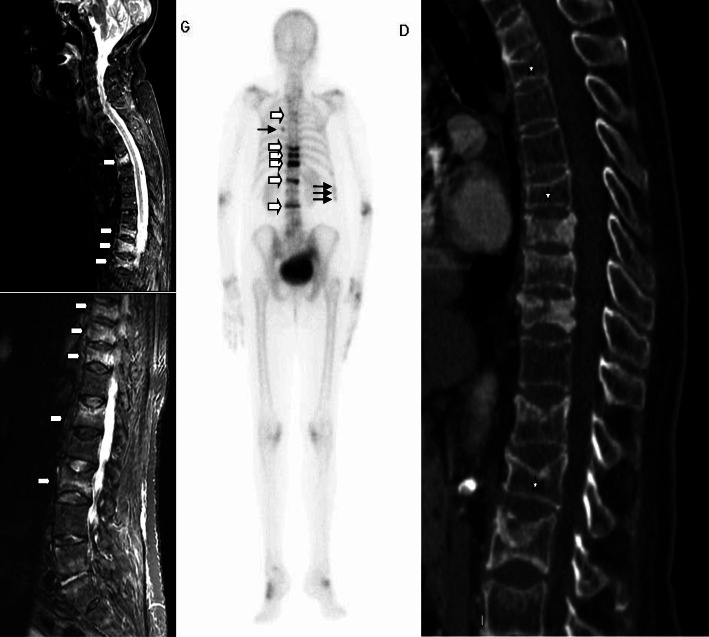
Bone scintigraphy, computed tomography scan, and magnetic resonance imaging in June 2014. Magnetic resonance imaging and bone scintigraphy confirmed that there were six recent vertebral fractures (fourth thoracic vertebra, ninth thoracic vertebra, tenth thoracic vertebra, 11th thoracic vertebra, first lumbar vertebra, and third lumbar vertebra): *small white arrows*. There were some costal fractures on bone scintigraphy: sixth posterior on the right side and laterally tenth, 11th, and 12th on the left side (*black arrows*). A computed tomography scan also showed old vertebral fractures (fifth thoracic vertebra, eighth thoracic vertebra, and second lumbar vertebra): *white stars*

In May 2014, our patient complained of acute intense spinal pain that resisted standard painkillers and required treatment with opiates. An evaluation was carried out in hospital in June 2014. Spinal X-rays revealed fractures of the fourth thoracic vertebra (T4; wedge, grade 2), T5 (biconcave, grade 3), T8 (wedge, grade 3), ninth thoracic vertebra (T9; crush, grade 3), tenth thoracic vertebra (T10; wedge, grade 1), 11th thoracic vertebra (T11; crush, grade 3), first lumbar vertebra (L1; biconcave, grade 1), L2 (wedge, grade 2), and third lumbar vertebra (L3; biconcave, grade 2) (Fig. 2). Bone scintigraphy revealed hypersignals in all these vertebrae except L2 and T5, and in several ribs. Magnetic resonance imaging (MRI) identified vertebra T4 in hypersignal on a T2-weighted sequence and hyposignal on a T1-weighted sequence, with no signs of infiltration or suspected lysis (Fig. 3). T9, T10, T11, L1, and L3 also showed hypersignal, and T5, T8, and L2 were older vertebral fractures with no bone marrow edema. Lumbar and dorsal pain remained severe throughout this period of exploration, justifying bed rest. Biological tests revealed hypercalcemia, with plasma concentrations of 2.83 mmol/l for calcium (normal range 2.13 to 2.65 mmol/l) (Fig. 1) and 1.06 mmol/l for phosphate (normal range 0.70 to 1.30 mmol/l), hypercalciuria (17.1 mmol/24 hours; normal range 1.5 to 6.2 mmol/24 hours), a 25(OH) vitamin D3 concentration of 15 ng/ml (normal range 30 to 60 ng/l), a PTH concentration of 41 pg/ml (normal range 15 to 65 ng/l), a C-reactive protein (CRP) concentration of 1.3 mg/l (normal values < 5 mg/l), normal protein electrophoresis with no Bence Jones proteinuria, and a plasma creatinine concentration of 44 μmol/l (normal range 45 to 84 μmol/l). Blood formula, and plasma concentrations of thyroid-stimulating hormone (TSH), parathyroid hormone-related peptide (PTHrp), cortisol and 1,25(OH)_2_D were normal. Carboxy-terminal collagen crosslink (CTX) levels were very high (2.09 μg/l; normal values < 0.43 μg/l), but were difficult to interpret in the context of vertebral fracture. DXA performed 1 year after the last injection of denosumab revealed BMD losses of 6.0% in our patient’s lumbar region and 2.9% in her total hip.

The association of fractures that are unusual for osteoporosis (T4), acute and persistent back pain, other rib fractures, and hypercalcemia were suggestive of a potential neoplasia, which led to systemic explorations, vertebral biopsy, and hyperparathyroidectomy and thyroidectomy (known goiter at the ultrasound exploration). A thoracic/abdominal/pelvic computed tomography (CT) scan showed only a heterogeneous multinodular goiter. Sesta-methoxy-isobutyl-isonitrile (MIBI) scintigraphy revealed a small area of fixation of the posterior lower right thyroid lobe and a lower lobe nodule displaying clear uptake. Fine-needle aspiration results were negative. A biopsy of the T4 was carried out under CT control and produced normal results. With hindsight, a gassy image of the upper facet of the T4 was suggestive of necrosis. A parathyroid neoplasia could have been evoked too. Surgery was performed at the end of July 2014 to remove the right upper parathyroid gland (13 × 10 × 2 mm; weight, 0.1 g), and histological analysis suggested nodular hyperplasia. Associated total thyroidectomy led to the detection of a dystrophic goiter with macrovesicular nodules and a 1 mm isthmic papillary microcarcinoma with no associated adenopathy.

Her pain was initially acute but of the mechanical type with a generally favorable outcome. Her calcaemia normalized the day after surgery: 2.44 mmol/l, with a plasma PTH concentration of 51.5 ng/l (normal range 15 to 65 ng/l). A new bone densitometry evaluation was carried out in October 2016, at which time bone losses of 15.7% for the lumbar region (T-score, − 2.9 SD) and 15.5% for the total left hip (T-score, − 2.3 SD) were recorded (Fig. 1). She continued to complain of disabling spinal pain. Her phosphorus and calcium evaluation results remained normal, as did her vitamin D levels, with the continuation of substitution treatment. Given the considerable decrease in BMD, she was placed on risedronate in September 2016, but this was badly tolerated. She was then placed on zoledronate (5 mg). Her calcaemia remained stable at 2.47 mmol/l (normal range 2.13 to 2.65 mmol/l). After two infusions (October 2016 and October 2017), a new DXA in August 2018 showed stabilization of the lumbar BMD (+ 0.5%) and a significant loss in the total hip BMD (− 8.5%). Her plasma calcium levels remained normal (2.60 mmol/l) and she did not have any new vertebral or peripheral fractures.

## Discussion and conclusions

This patient, who was included in the initial FREEDOM protocol [18], had benefited from denosumab treatment, with no new vertebral fractures in the last 3 years of treatment and an increase in BMD to values exceeding − 1.6 SD, with no secondary effects. However, 10 months after stopping the treatment, a cascade of vertebral fractures, some in unusual locations (T4, although this can be possible when there are multiple lower vertebral fractures), and multiple rib fractures in a context of hypercalcemia suggested possible malignancy. A complete evaluation, with systemic, biological, and biopsy (T4) analyses, ruled out this hypothesis. Histological analysis of the tissue removed during the parathyroid intervention revealed hyperplasia, but no adenoma. The hypothesis of coincidental hyperparathyroidism had to be considered. Before the treatment with denosumab, our patient’s calcaemia and PTH levels were normal. Based on retrospective analyses of calcaemia results, we concluded that the increase in calcaemia became abnormal after 8 years of denosumab treatment. Hyperparathyroidism could have appeared during the denosumab treatment phase with no obvious link between the two occurrences; however, there were no clear increases in calcaemia during the first 8 years, and hypercalcemia was markedly aggravated by stopping denosumab. The link between hyperparathyroidism and denosumab was the subject of a recent publication [19]. However, the hyperparathyroidism described occurred rapidly after a single injection of denosumab, with a 1.3-fold increase in PTH levels at 1 week, normalization at 2 months, and normal calcaemia throughout [19]. This case resolved within a year. In our case, calcaemia was high after 8 years of treatment. No PTH determinations were carried out in parallel during this period. It is therefore difficult to determine the date of onset of the hyperparathyroidism, as the denosumab treatment may have masked the hypercalcemia. Similarly, as our patient underwent surgery, it is impossible to know what spontaneous course it would have taken.

Two similar cases of hypercalcemia during the rebound effect after stopping treatment with denosumab have been reported: one with low PTH levels and a spontaneously favorable outcome over several months [7], and the other after a high dosage of denosumab (120 mg quarterly) [14]. The excessive bone remodeling observed in the absence of associated fractures may have been due to major bone reabsorption potentially accounting for the hypercalcemia, as in immobilization-related osteoporosis. The high hypercalcemia observed is consistent with this hypothesis. Alendronate was administered and the patient’s plasma calcium concentrations returned to normal values within 5 months. In contrast, our patient had a PTH concentration that was well controlled and not high, and displayed rapid normalization (within 24 hours) of calcaemia after the intervention. Normal phosphate, mid-upper calcaemia, and normal creatinine were not in favor of tertiary hyperparathyroidism. However, the hyperparathyroidism described occurred rapidly, after a single injection of denosumab, with a 22-fold increase in intact PTH (iPTH) levels at 1–8 weeks and normal calcaemia throughout in a case report [15]. This dramatic increase in iPTH resolved spontaneously within a year. Given the surgical intervention carried out on our patient, we cannot determine what the natural course of this hypercalcemia might have been, in the same way that half the cases of hyperparathyroidism in kidney transplant recipients resolve within 1 year for example [20]. The surgery was carried out because we were concerned that our patient might be suffering from a parathyroid carcinoma.

This rebound effect has been reported after stopping denosumab administered at an oncological dosage (120 mg monthly for 9 months) [21]. Seven non-malignant vertebral crushes were observed 15 months after the last injection of denosumab. At lower dosages (denosumab 60 mg half-yearly for 5 years), 15 patients with breast cancer receiving aromatase inhibitors developed 60 vertebral crushes after stopping denosumab [22]. The risk of vertebral fracture was higher if the treatments were longer and if the patients had pre-existing osteoporosis.

Eight other cases of hypercalcemia have been reported, in children. Denosumab was administered in these cases for giant-cell tumors, fibrous dysplasia, brittle bone disease, and juvenile Paget’s disease [7–13]. The hypercalcemia occurred early (2 to 5 months after the denosumab injection) and was sometimes severe (plasma calcium concentrations of up to 3.8 mmol/l), but it occurred in a context of high doses and bone remodeling not comparable with the context in adults and the rebound effect. The hypercalcemia regressed over a few months, either spontaneously or on zoledronate. Reactional bone hyper-reabsorption was again suggested. This hyper-reabsorption seems to be related to the release of receptor activator of nuclear factor kappa-B ligand (RANK-L), with high crosslinked carboxy-terminal telopeptide of type 1 collagen (CTX-1), and low Dickkopf-1 (DKK1) and sclerostin [23]. Osteocytes are known to be the principal source of RANK-L [24]. We have suggested that osteocytes may undergo apoptosis during this rebound effect when the treatment with denosumab is stopped, potentially accounting for the necrosis and strong hyper-reabsorption reported in certain patients presenting this rebound effect [16].

The discovery of a thyroid papillary microcarcinoma appears to have been fortuitous in this case. Other studies have shown that denosumab may not only decrease the frequency of bone tumor events, but even have a direct or indirect antitumoral effect [25]. However, a meta-analysis comparing denosumab and zoledronate and including four randomized trials (7379 patients) found a significantly higher risk of primary neoplasia, with a cumulative annual incidence of 1.1% on denosumab, versus 0.6% on zoledronate [17]. No particular cancer type profile was identified, but the number of cases was small (*n* = 54). The cumulative doses in our patient amounted to approximately 1 g of denosumab, corresponding to 8 months at the dose of 120 mg/month prescribed to prevent secondary bone tumor complications. It is not possible to draw any firm conclusions concerning our case, as goiter is itself a risk factor for thyroid cancer, with a poorly defined incidence of between 4 and 11% for cancer in patients undergoing surgery for goiter [26].

In conclusion, it is important to be aware of this rebound effect with strong bone hyper-reabsorption in certain patients after stopping treatment with denosumab. Several explorations to eliminate a neoplasm could be avoided with knowledge of these cases. It can take several different forms: a simple loss of the BMD gained on the treatment, vertebral fractures, or transient hypercalcemia, which in this context may raise concerns of, or simulate, malignant bone diseases or hyperparathyroidism, as in our case. The rebound after stopping treatment with denosumab makes it necessary to check calcaemia and CTX early, before the risk of further vertebral fractures. DXA will be carried out at the end of the denosumab sequence, but an early new comparative DXA would be less sensitive than CTX dosage. We can propose CTX and calcaemia 6 months after the last injection of denosumab, as a reference, and then 2 or 3 months later. A marked increase will require prevention. The most appropriate therapeutic approach remains unclear. The effects of gradually decreasing the dose of denosumab have not yet been reported. Bisphosphonates seem to be only partially effective according to published preliminary results [27].

## Data Availability

All original data are available (corresponding author).

## Abbreviations

BMD: Bone mineral density
CRP: C-reactive protein
CT: Computed tomography
CTX: Carboxy-terminal collagen crosslink
CTX-1: Crosslinked carboxy-terminal telopeptide of type 1 collagen
DKK1: Dickkopf-1
DXA: Dual X-ray absorptiometry
iPTH: Intact parathyroid hormone
L1: First lumbar vertebra
L2: Second lumbar vertebra
L3: Third lumbar vertebra
MRI: Magnetic resonance imaging
PTH: Parathyroid hormone
PTHrp: Parathyroid hormone-related peptide
RANK-L: Receptor activator of nuclear factor kappa-B ligand
SD: Standard deviation
T4: Fourth thoracic vertebra
T5: Fifth thoracic vertebra
T8: Eighth thoracic vertebra
T9: Ninth thoracic vertebra
T10: Tenth thoracic vertebra
T11: 11th thoracic vertebra
TSH: Thyroid-stimulating hormone
MIBI: Methoxy-isobutyl-isonitrile
